# mtIF3 is locally translated in axons and regulates mitochondrial translation for axonal growth

**DOI:** 10.1186/s12915-021-01215-w

**Published:** 2022-01-07

**Authors:** Soyeon Lee, Dongkeun Park, Chunghun Lim, Jae-Ick Kim, Kyung-Tai Min

**Affiliations:** 1 Department of Biological Sciences, Ulsan National Institute of Science and Technology (UNIST), Ulsan, 44919 Republic of Korea; 2 National Creative Research Initiative Center for Proteostasis, Ulsan National Institute of Science and Technology (UNIST), Ulsan, 44919 Republic of Korea

**Keywords:** Local translation, Mitochondrial translation, Axon development, Mitochondria, Bimolecular fluorescence complementation

## Abstract

**Background:**

The establishment and maintenance of functional neural connections relies on appropriate distribution and localization of mitochondria in neurites, as these organelles provide essential energy and metabolites. In particular, mitochondria are transported to axons and support local energy production to maintain energy-demanding neuronal processes including axon branching, growth, and regeneration. Additionally, local protein synthesis is required for structural and functional changes in axons, with nuclear-encoded mitochondrial mRNAs having been found localized in axons. However, it remains unclear whether these mRNAs are locally translated and whether the potential translated mitochondrial proteins are involved in the regulation of mitochondrial functions in axons. Here, we aim to further understand the purpose of such compartmentalization by focusing on the role of mitochondrial initiation factor 3 (mtIF3), whose nuclear-encoded transcripts have been shown to be present in axonal growth cones.

**Results:**

We demonstrate that brain-derived neurotrophic factor (BDNF) induces local translation of mtIF3 mRNA in axonal growth cones. Subsequently, mtIF3 protein is translocated into axonal mitochondria and promotes mitochondrial translation as assessed by our newly developed bimolecular fluorescence complementation sensor for the assembly of mitochondrial ribosomes. We further show that BDNF-induced axonal growth requires mtIF3-dependent mitochondrial translation in distal axons.

**Conclusion:**

We describe a previously unknown function of mitochondrial initiation factor 3 (mtIF3) in axonal protein synthesis and development. These findings provide insight into the way neurons adaptively control mitochondrial physiology and axonal development via local mtIF3 translation.

**Supplementary Information:**

The online version contains supplementary material available at 10.1186/s12915-021-01215-w.

## Background

Mitochondrial oxidative phosphorylation complexes primarily generate ATP essential for cellular function in neuronal cell bodies and neurites. In fact, mitochondria are transported to axons and produce local energy for axon branching, growth cone formation, and axon growth [1–3]. The localized mitochondria also play a significant role in facilitating axonal regeneration after injury [4–6]. Thus, rapid ATP synthesis in response to local energy demand is likely crucial, particularly for polarized neuronal function.

Although active mitochondrial transport to axonal tip has been shown to support local energy needs [7, 8], this may not be sufficient to explain how neurons adaptively regulate mitochondrial function in axons [9]. Hence, we reason that additional mechanisms, such as local synthesis of mitochondrial proteins, should contribute to the functional control of axonal mitochondria. Most mitochondrial genes are nuclear-encoded, and once transcribed, their mRNA translation generally occurs in the cell bodies. On the other hand, previous studies have revealed that transcripts of the nuclear-encoded mitochondrial genes can be locally translated in distal axons [10–14]. Nonetheless, it is still elusive whether any local synthesis of the nuclear-encoded mitochondrial proteins governs mitochondrial function in axon growth cone.

In mammalian cells, mitochondria have only two mitochondrial translation initiation factors, mtIF2 and mtIF3 [15]. Interestingly, translatome analyses have revealed mtIF3 translation in axon growth cone [11], suggesting a possible role of local mtIF3 synthesis in regulating axonal mitochondrial translation. mtIF3 regulates the dynamics of ribosome association on mitochondrial mRNAs. mtIF3 catalyzes the dissociation of mitochondrial ribosomes (mitoribosomes) into large and small subunits while blocking any premature binding of the large subunit [16, 17]. mtIF2 and N-formylmethionine-tRNA bind weakly to the small subunit in the absence of mRNA, but mtIF3 facilitates mRNA binding to the small subunit so that a start codon can be correctly positioned at P-site [15, 17].

Given the critical role of mtIF3 in mitochondrial translation initiation [18], it is plausible that locally synthesized mtIF3 may regulate mitochondrial translation in developing axons to support ATP synthesis and relevant physiology. Studies on mitochondrial translation in live cells, however, have been hampered by a lack of appropriate tools. Here, we have developed a molecular sensor that visualizes mitochondrial translation activity using the bimolecular fluorescence complementation (BiFC) between a specific pair of mitoribosome proteins. In conjunction with additional transgenic reporters for functional imaging, this new tool has led us to test the hypothesis above and validate the significance of local mtIF3 translation in mitochondrial physiology and axonal growth.

## Results

### BDNF induces local protein synthesis of mtIF3 in axon growth cone

**Fig. 1:**
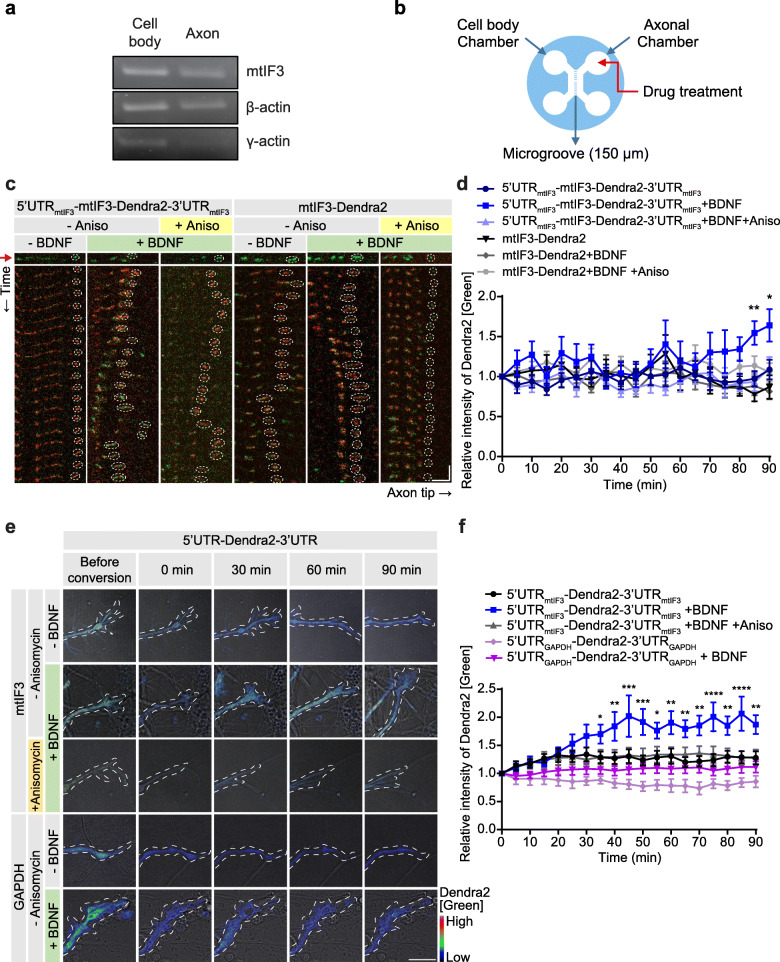
BDNF induces local protein synthesis of mtIF3 in axon growth cone. **a** mtIF3 mRNAs were detected in both cell bodies and axons of primary hippocampal neurons at DIV4. RNA samples were purified from the isolated lysates of cell bodies and axons. RT-PCR was performed using each pair of gene-specific primers. β-actin existing in both lysates and γ-actin detected in cell bodies indicate that both cell bodies and axons were fractionated without contamination [19]. **b** Primary hippocampal neurons were seeded into the cell body chamber. Axons reached the other side of the device through the microgroove. To locally stimulate axons, the only axonal chamber was treated with drugs. **c** Kymographs of newly synthesized mtIF3-Dendra2 fusion proteins in axons. Neurons were cultured on microfluidic devices and transfected with an expression vector for the mtIF3-Dendra2 protein at DIV3. Fluorescent intensity of mtIF3-Dendra2 fusion was measured in mitochondrial areas at DIV4 (horizontal scale bar, 5 μm; vertical scale bar, 5 min). The red arrow indicates images before photoconversion. The existing mtIF3-Dendra2 was photo-converted from green to red over the 20 μm path from the axon tip. Green signal is non-photoconverted mtIF3-Dendra2 fluorescence and red signal is photo-converted fluorescence. Recovered green signal from existing red mitochondria was analyzed. Images were then taken at 5-min intervals for 90 min. BDNF (30 ng/ml), and anisomycin (20 μM) were added to the axonal chamber at a 0-min timepoint. **d** Quantification of newly synthesized mtIF3-Dendra2 proteins. The green fluorescence was measured from mitochondria at the very end of the axonal tip. The relative intensity was calculated by normalizing the values at each time point to the value at a 0-min time point. Data represent mean ± SEM (*N* = 3 replicates and *n* = 6–9 axons). *P < 0.05, **P < 0.01 as determined by two-way repeated-measures ANOVA with Holm-Sidak’s multiple comparisons test. Asterisk indicates statistical significance from 5′UTR_mtIF3_-mtIF3-Dendra2-3′UTR_mtIF3_ with BDNF treatment for 5′UTR_mtIF3_-mtIF3-Dendra2-3′UTR_mtIF3_ without treatment or with BDNF and anisomycin treatments. No statistical significance was observed among mtIF3-Dendra2 groups without UTRs. **e** Pseudo-color images of locally synthesized Dendra2 reporter in axonal tip (scale bar, 10 μm). The experiment was performed similarly as in panel c. **f** Quantification of newly synthesized Dendra2 reporters in axonal tip after drug treatment. Data represent mean ± SEM (*N* = 3 replicates and *n* = 6–10 axons). *P < 0.05, **P < 0.01, ***P < 0.001, ****P < 0.0001 as determined by two-way repeated-measures ANOVA with Holm-Sidak’s multiple comparisons test. Asterisk indicates statistical significance from 5′UTR_mtIF3_-Dendra2-3′UTR_mtIF3_ with BDNF treatment for 5′UTR_mtIF3_-Dendra2-3′UTR_mtIF3_ without treatment or BDNF and anisomycin treatments. No statistical significance was observed between 5′UTR_GAPDH_-Dendra2-3′UTR_GAPDH_ groups

We first confirmed that mtIF3 mRNAs were present in both cell bodies and axons of primary hippocampal neurons (Fig. 1a), consistent with a previous report [11]. To examine whether locally translated mtIF3 proteins translocate into mitochondria, we generated a transgene that expresses fluorescent mtIF3 proteins fused to photo-convertible Dendra2 along with mtIF3 untranslated regions (UTRs) (5′UTR_mtIF3_-mtIF3-Dendra2-3′UTR_mtIF3_). As expected, the coding sequence (CDS) of mtIF3 led to mitochondrial localization of mtIF3-Dendra2 fusion in primary hippocampal neurons likely due to its mitochondrial targeting sequence [20] (Additional file 1: Fig. S1a, b). We further employed a microfluidic device to separate the cell bodies and axons of primary hippocampal neurons into two distinct chambers [21] (Fig. 1b) and assess local effects in axonal growth cone. The fluorescent mtIF3-Dendra2 proteins at the tip of axons were irreversibly photo-switched from green to red using 405 nm illumination, and then newly translated mtIF3-Dendra2 proteins with green fluorescence were measured by analyzing time-lapse images taken every 5 min for 90 min. Several studies have suggested that many nuclear-encoded mitochondrial proteins might be synthesized in response to local energy demand [11–14]. This prompted us to examine whether BDNF treatment in the axonal chamber of microfluidic devices enhances the local translation of the mtIF3-Dendra2 fusion reporter, thereby leading to its mitochondrial translocation in axonal growth cone. We performed kymograph analyses over ~ 20-μm distal axons and found that local BDNF treatment indeed elevated the newly synthesized mtIF3-Dendra2 signals in axonal mitochondria of 5′UTR_mtIF3_-mtIF3-Dendra2-3′UTR_mtIF3_ transfected cells (Fig. 1c, d; Additional file 1: Fig. S1c). A general translation inhibitor, anisomycin, blocked de novo synthesis of the reporter protein, validating that mtIF3 protein is translationally upregulated by BDNF treatment. Most of the axonal proteins are synthesized in the cell body and actively transported to axons [22]. To exclude the possibility that newly translated mtIF3-Dendra2 is transported from the cell body upon BDNF stimulation, we generated the construct of mtIF3-Dendra2 without UTRs (mtIF3-Dendra2). Given that mtIF3 3′UTR contains a consensus motif (CTCCCATC) shared by axon-enriched mRNAs [11], it is unlikely that reporter mRNAs lacking the mtIF3 UTRs are actively transported to axon growth cone. Deletion of UTRs indeed disturbed the upregulation of newly generated mtIF3-Dendra2 signals upon BDNF treatment while the protein distribution of mtIF3-Dendra2 without UTRs was identical with that from 5′UTR_mtIF3_-mtIF3-Dendra2-3′UTR_mtIF3_ (Fig. 1c, d; Additional file 1: Fig. S1b, c). These data support that BDNF treatment triggers the local synthesis of mtIF3 proteins in axon growth cone via the mtIF3 UTRs.

To further validate whether mtIF3 UTRs are sufficient for BDNF-induced local translation, we generated two additional translation reporters encoding the fluorescent Dendra2 along with UTRs from mtIF3 (5′UTR_mtIF3_-Dendra2-3′UTR_mtIF3_) or GAPDH (5′UTR_GAPDH_-Dendra2-3′UTR_GAPDH_). It has been reported that the translation of GAPDH is not induced by BDNF in the axon [23], while the N-terminal palmitoylation sequence in the reporter protein served to limit its free diffusion from the cell body [5, 24]. BDNF treatment in an axonal chamber of a microfluidic device gradually increased the fluorescence of newly synthesized Dendra2 from the mtIF3 UTR reporter at the axonal tip, whereas anisomycin treatment suppressed it (Fig. 1e, f). We detected no significant changes in the fluorescence from the control GAPDH UTR reporter upon BDNF treatment. Notably, we observed that after BDNF treatment, the signal intensity of Dendra2 without CDS of mtIF3 increases faster than that of Dendra2 with CDS of mtIF3. It is conceivable that conjugation of mtIF3 CDS to Dendra2 could delay the import of proteins into mitochondrial matrix, protein folding, accumulation of proteins, and maturation of fluorescent proteins. Thus, these aforementioned factors might collectively have contributed to differential temporal dynamics of Dendra2 signal between mtIF3-Dendra2 and Dendra2 upon BDNF treatment. Together, these results suggest that mtIF3 proteins are locally synthesized in axon growth cones in response to BDNF. In addition, mtIF3 UTRs likely support the axonal transport of mtIF3 mRNAs and their BDNF-induced translation in distal axons.

### Mito-riboBiFC detects translation-dependent assembly of mitoribosomes

**Fig. 2:**
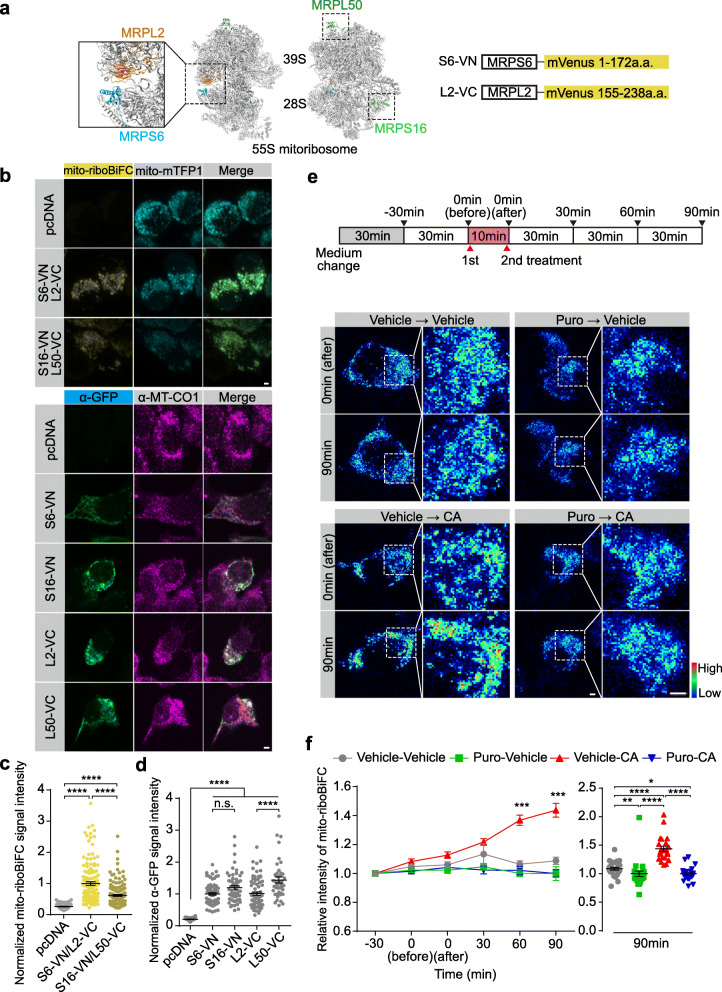
Mito-riboBiFC detects translation-dependent assembly of mitoribosomes. **a** Schematic design of mito-riboBiFC. Mitochondrial ribosomal proteins MRPL2 and MRPS6 were used as a BiFC pair for the mito-riboBiFC and illustrated with porcine 55S mitoribosome cryo-EM structure [28]. MRPS16 and MRPL50 served as a negative control. **b** Representative images of mito-riboBiFC (top) and mVenus-detectable GFP antibody staining (bottom) in Neuro2A cells. Mito-mTFP1 and MT-CO1 were used as mitochondrial markers (scale bar, 2 μm). **c** Quantification of the fluorescent mito-riboBiFC signals in panel b. Mito-riboBiFC signals were normalized to the average of fluorescent signals in S6-VN/L2-VC group. Data represent mean ± SEM (*N* = 3 replicates and *n* = 100–143 cells). ****P < 0.0001, as determined by aligned ranks transformation ANOVA with Wilcoxon rank-sum test. **d** Quantification of signal intensity of GFP antibody staining in **b**. GFP signals were normalized to the average of fluorescent signals in S6-VN group. Data represent mean ± SEM (*N* = 3 replicates and *n* = 41–76 cells). n.s., not significant; ****P < 0.0001, as determined by aligned ranks transformation ANOVA with Wilcoxon rank-sum test. **e** Pseudo-color images of mito-riboBiFC after sequential treatment of puromycin (Puro) and chloramphenicol (CA) (scale bar, 2 μm). **f** Quantification of the mito-riboBiFC signals in **e**. Line plot shows the intensity changes of mito-riboBiFC. A Vehicle-CA group was compared with other groups at each time point for the statistical test (left panel). Dot plot displays the relative intensity of the mito-riboBiFC 90 min after CA treatment (1st vehicle, water; 2nd vehicle, ethanol). Data represent mean ± SEM (*N* = 3 replicates and *n* = 22–26 cells). Aligned ranks transformation ANOVA detected significant interaction effects of Puro and CA on the mito-riboBiFC intensity at the 90-min time point (*P* < 0.0001). **P < 0.01, ****P < 0.0001, as determined by Wilcoxon signed-rank test (left panel) or Wilcoxon rank-sum test (right panel)

We hypothesized that BDNF-induced local translation of mtIF3 proteins might be involved in regulating mitochondrial translation in developing axon tips. To overcome possible limitations in the biochemical assessment of mitochondrial translation in distal axons, we devised a new strategy to visualize mitochondrial translation in live cells using BiFC [25] which was previously used for the visualization of cytoplasmic ribosomal subunit joining [26]. This was based on the physical proximity of mitochondrial ribosomal protein L2 (MRPL2) and mitochondrial ribosomal protein S6 (MRPS6) at the inter-subunit bridge of 55S mitoribosome [27] (Fig. 2a). We took advantage of this adjacent localization of the two MRPs as a BiFC pair to visualize mitoribosome assembly during translation. In detail, we split a fluorescent protein mVenus into N-terminal (VN, 1-172 amino acids) and C-terminal fragments (VC, 155-238 amino acids). Then, we fused these mVenus fragments to the C-termini of MRPS6 (S6-VN) and MRPL2 (L2-VC), respectively. Short peptide linkers [26] were inserted between MRPs and mVenus fragments to increase the flexibility of MRP-mVenus fusion proteins (S6-VN, L2-VC), minimize non-specific interactions, and facilitate the proper assembly of mitoribosomes. The co-expression of S6-VN and L2-VC in Neuro2A cells generated mVenus fluorescent signals exclusively in mitochondria (Fig. 2b). On the other hand, another pair of MRPs positioned distantly from each other in mitoribosomes showed relatively weak fluorescent signals (Fig. 2a–c, MRPS16 and MRPL50). It is unlikely that the latter pair displayed weak BiFC due to their low expression levels (Fig. 2d). Lack of mito-riboBiFC signals in cells only expressing the fluorescent mitochondrial marker (i.e., mito-mTFP1) confirmed no bleed-through between the two imaging channels (Fig. 2b, c).

Unlike cytoplasmic ribosomes, two mitoribosome subunits could be assembled in the absence of translating mRNAs [15]. Moreover, it has been reported that non-specific assembly of the mVenus fragments could lead to detectable BiFC [29]. We thus asked if the BiFC signals from the S6-VN and L2-VC pair would actually depend on the translation of mitochondrial mRNAs. When translating ribosome subunits were dissociated from mRNAs by puromycin, the BiFC signals were reduced only by 8% over the 90-min period compared to the vehicle control (Fig. 2e, f). This observation may suggest that 8% of the BiFC signals would represent translation-dependent complementation of S6-VN and L2-VC in mitochondria, although we cannot rule out possible puromycin effects on the translation of S6-VN and L2-VC proteins per se.

The average cytoplasmic translation rate is six amino acids per second [30], and the longest transcript mt-ND5 mRNA is 1824 bp. In contrast, the fluorescence signals from the BiFC pair become detectable 10 min after complementation [31]. Considering that the translation of individual mitochondrial mRNAs could be completed in less than 2 min, it is likely that the translating mitoribosome dissociates from mRNAs before the chromophore maturation. We thus reasoned that the stabilization of translating mitoribosome would better visualize their mRNA-dependent emission of the complemented fluorescence signals. To this end, we employed chloramphenicol (CA) that selectively stalls mitoribosomes by inhibiting the peptide bond formation at the A-site [32, 33]. CA markedly increased the BiFC signals 60 min after treatment, whereas puromycin pre-treatment blocked the CA effects (Fig. 2e, f). Accordingly, we concluded that CA-induced BiFC signals would represent actively translating mitoribosome and designated our new tool for visualizing mitochondrial translation as mito-riboBiFC (Additional File 2).

### Locally synthesized mtIF3 promotes mitochondrial translation in axon growth cone

**Fig. 3:**
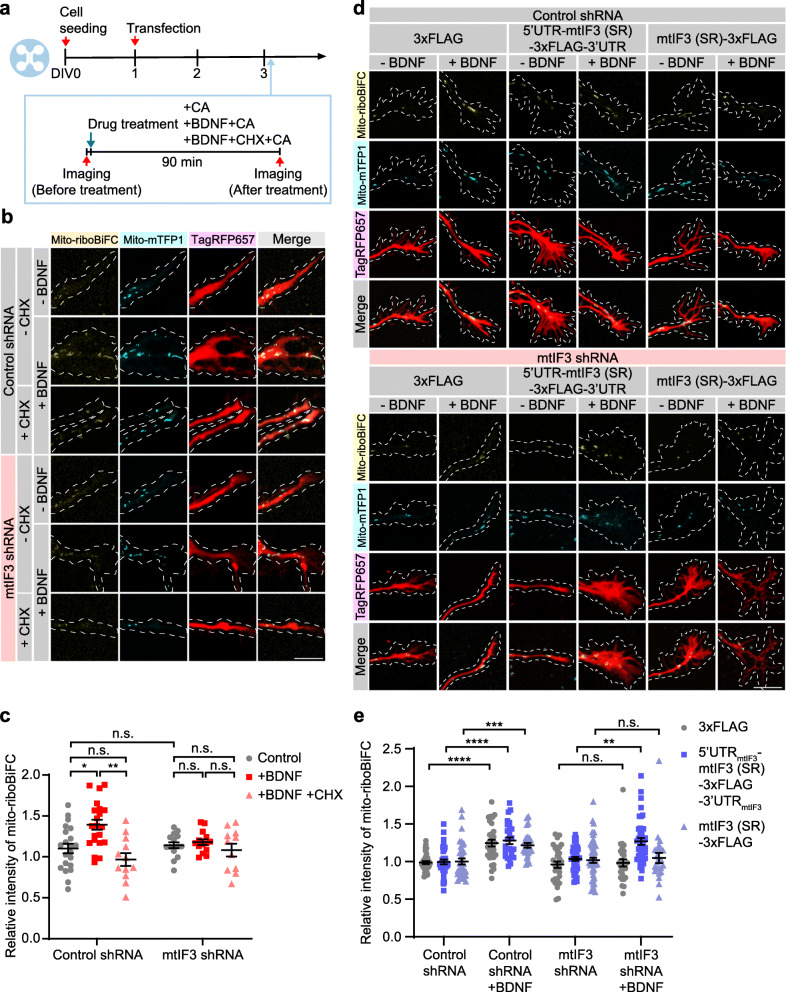
Local protein synthesis of mtIF3 is necessary for mitochondrial translation in axonal growth cone. **a** Timeline for mito-riboBiFC experiments. After cell seeding, shRNA and overexpression vectors were transfected at DIV1. At DIV3, images were sequentially taken before and after drug treatment. BDNF, CHX, and CA were treated simultaneously. Drugs in the axonal chamber were treated for 90 min. **b**, **c** Visualization of mitochondrial translation in mtIF3-depleted axon growth cones by mito-riboBiFC. Mitochondria were marked by mitochondria-targeted mTFP1. Transfection of shRNA was confirmed by TagRFP657 expression (scale bar, 10 μm). Mito-riboBiFC signals from mitochondrial staining by mito-mTFP1 were quantified, and the relative intensity of BiFC increase upon drug treatment was measured. Five mitochondria per axon were analyzed. Data represent mean ± SEM (*N* = 3 replicates and *n* = 11–21 axons). Aligned ranks transformation ANOVA detected significant interaction effects of mtIF3 depletion and BDNF on the increase of relative BiFC intensity (*P* = 0.0180). n.s., not significant; *P < 0.05, **P < 0.01 as determined by Wilcoxon rank-sum test. **d**, **e** Representative images of mito-riboBiFC in axon growth cones expressing shRNA and mtIF3 (SR) (scale bar, 10 μm). Mito-riboBiFC signals from mitochondrial staining mito-mTFP1 were analyzed before and after CA treatment. Five mitochondria per axon were analyzed. Data represent mean ± SEM (*N* = 6 replicates and *n* = 24–54 axons). Aligned ranks transformation ANOVA detected significant interactions of mtIF3 (SR) overexpression and BDNF on mito-riboBiFC in mtIF3 shRNA groups (but not in control shRNA groups) (*P* = 0.0123). n.s., not significant; **P < 0.01, ***P < 0.001, ****P < 0.0001 as determined by Aligned ranks transformation ANOVA with Wilcoxon rank-sum test

To test whether locally synthesized mtIF3 facilitates mitochondrial translation in axon growth cone, we manipulated mtIF3 expression by transient transfections and examined their effects on the mito-riboBiFC signals in axon growth cones. We first confirmed mtIF3 depletion using short hairpin RNA (shRNA) in NIH/3 T3 cells (Additional File 3: Fig. S3a, b). Next, we cultured hippocampal neurons on a microfluidic device to separate axons from cell bodies [21] and treated all the drugs only in the axonal channel to induce or block local translation (Fig. 3a). Given that anisomycin is known to inhibit not only cytosolic translation, but also possibly mitochondrial translation [34], we used cycloheximide (CHX) to inhibit only cytosolic translation. The degree of mitochondrial translation was subsequently quantified by CA-induced changes in the BiFC intensity (Additional File 2). Notably, neurons expressing control shRNA exhibited BDNF-induced mito-riboBiFC signals in axon growth cone. However, the treatment of CHX in the axonal channel completely blocked the increment of mito-riboBiFC signals upon BDNF treatment, indicating that BDNF-induced local protein synthesis promotes mitochondrial translation. Importantly, mtIF3 depletion abolished the BDNF-induced mito-riboBiFC signals (Fig. 3b, c). Given that BDNF induces mtIF3 translation in distal axons, these results together suggest that local mtIF3 synthesis might be necessary for facilitating mitochondrial translation upon BDNF treatment, although shRNA-mediated depletion could lower mtIF3 levels in both cell body and axon.

To assess whether local translation of mtIF3 is indeed required for promoting mitochondrial translation upon BDNF treatment, we generated mtIF3 transgenes that encode a shRNA-resistant (SR) mtIF3 CDS with or without mtIF3 UTRs (Additional File 3: Fig. S3c-e). The mtIF3 overexpression vectors also included an independent expression cassette for the fluorescent mito-mTFP1 protein to visualize mitochondria in transfected cells for live-cell imaging (Additional File 3: Fig. S3f). Primary hippocampal neurons were co-transfected with expression vectors for shRNA, mtIF3 (SR), and mito-riboBiFC, and we examined if the mtIF3 transgene could rescue mtIF3 depletion phenotypes in mitochondrial translation. mtIF3 (SR) overexpression in control neurons negligibly affected mito-riboBiFC signals regardless of BDNF treatment (Fig. 3d, e). However, it specifically restored BDNF-induced mitochondrial translation in mtIF3-depleted neurons. The rescue required mtIF3 UTRs since mtIF3 (SR) overexpression from the transgene lacking mtIF3 UTRs failed to rescue the mtIF3-depletion phenotype despite high levels of mtIF3 overexpression (Fig. 3d, e; Additional File 3: Fig. S3e). Considering that mtIF3 UTRs mediate BDNF-induced reporter expression in distal axons (Fig. 1e, f), these results further support that local translation of mtIF3 facilitates mitochondrial translation upon BDNF treatment in axonal growth cone.

### mtIF3-dependent mitochondrial translation elevates ATP generation in growing axons

**Fig. 4:**
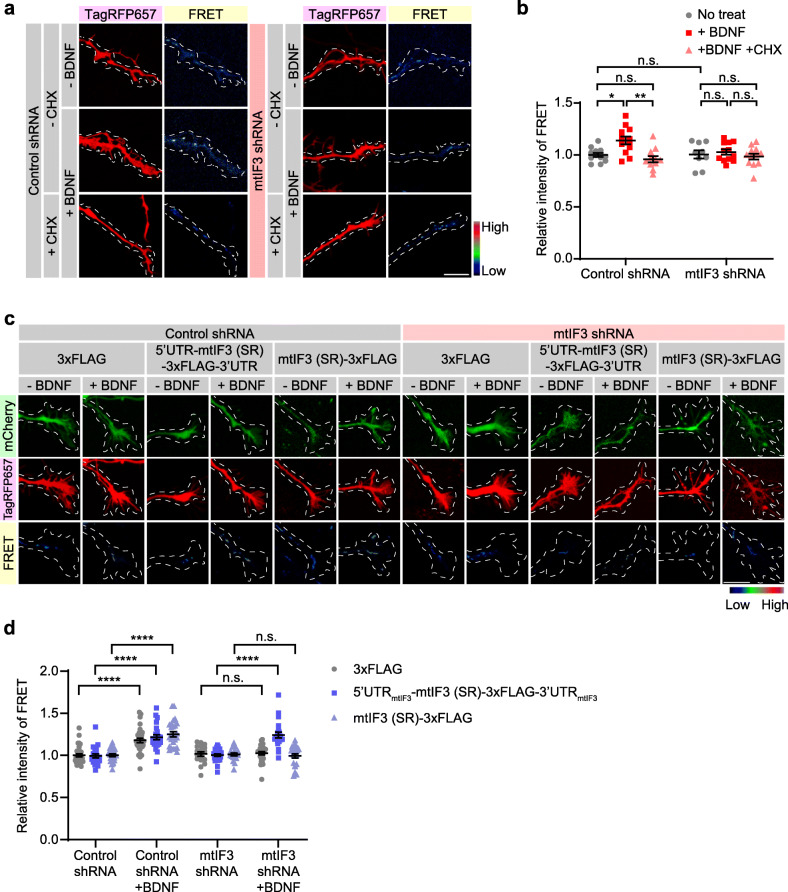
mtIF3-dependent mitochondrial translation elevates ATP generation in axonal growth cone. **a**, **b** ATP levels in mtIF3-depleted axonal mitochondria were measured using genetically encoded ATP indicator mito-ATeam1.03. After cell seeding, shRNA and mito-ATeam1.03 vectors were transfected at DIV1. At DIV3, images were sequentially taken before and after drug treatment. BDNF and CHX were treated simultaneously. Drugs in the axonal chamber were treated for 90 min. FRET signals were shown in the pseudo-color image. Expression of shRNA was confirmed by TagRFP657 expression (scale bar, 10 μm). FRET signals were measured by comparing the ratio before and after chemical treatments. Five mitochondria per axon were analyzed. Data represent mean ± SEM (*N* = 4 replicates and *n* = 9–12 axons). n.s., not significant; *P < 0.05, **P < 0.01 as determined by two-way ANOVA with Holm-Sidak’s multiple comparisons test. **c**, **d** Relative ATP levels in axonal mitochondria of neurons expressing shRNA and mtIF3 (SR). After cell seeding, shRNA, overexpression, and mito-ATeam1.03 vectors were transfected at DIV1. At DIV3, images were sequentially taken before and after BDNF treatment. BDNF in the axonal chamber was treated for 90 min. Pseudo-color shows ATP levels in mitochondria. mCherry and TagRFP657 expression confirms expression of mtIF3 (SR) and shRNA, respectively (scale bar, 10 μm). The increase of relative FRET signals was calculated by comparing before and after chemical treatments. Five mitochondria per axon were analyzed. Data represent mean ± SEM (*N* = 5 replicates and *n* = 17–34 axons). Aligned ranks transformation ANOVA detected significant interactions of mtIF3 (SR) overexpression and BDNF on the FRET signals in mtIF3 shRNA groups (but not in control shRNA groups) (*P* < 0.0001). n.s., not significant; ****P < 0.0001 as determined by Aligned ranks transformation ANOVA with Wilcoxon rank-sum test

Next, we questioned whether locally translated mtIF3 would control mitochondrial physiology in developing axons. To this end, we employed mito-ATeam1.03, a genetically encoded FRET (Fluorescence Resonance Energy Transfer) sensor for mitochondrial ATP [35]. CA treatment to primary hippocampal neurons expressing mito-ATeam1.03 reduced the intensity of the FRET signals, indicating that mitochondrial ATP generation requires mitochondrial translation (Additional File 4). We then cultured primary hippocampal neurons in a microfluidic device and assessed the local effects of BDNF and translation inhibitors on the mito-ATeam1.03 signals in the axonal channel. BDNF treatment elevated mitochondrial ATP levels in axon growth cone, whereas blocking local translation by CHX nullified this BDNF effect (Fig. 4a, b). mtIF3 depletion also blunted BDNF-induced increase in mitochondrial ATP levels, yet it negligibly affected the baseline ATP levels (Fig. 4a, b). To determine whether locally synthesized mtIF3 is essential for mitochondrial ATP generation, mCherry expressing mtIF3 (SR) vectors (Additional File 3: Fig. S3g) were co-transfected with mito-ATeam1.03. We observed the rescue effects of overexpressing mtIF3 (SR) with UTRs on mitochondrial ATP levels in distal axons under BDNF treatment (Fig. 4c, d), consistent with the effects of locally synthesized mtIF3 on mitochondrial translation in axon growth cone (Fig. 3d, e). These results support our model that BDNF-induced local synthesis of mtIF3 promotes mitochondrial translation and elevates ATP generation in axonal mitochondria, thereby fulfilling local energy demand in developing axons.

### Axonal development requires mtIF3-dependent mitochondrial translation in growing axons

**Fig. 5:**
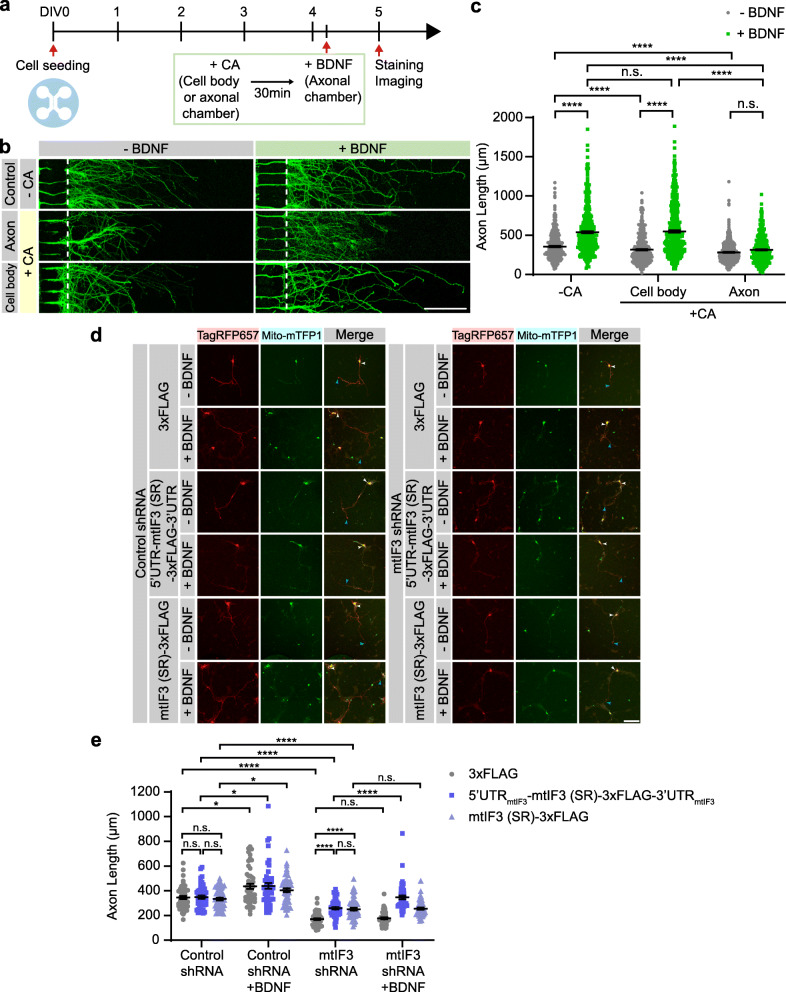
Axonal development requires mtIF3-dependent mitochondrial translation in growing axons. **a** Timeline for the experiment. Primary hippocampal neurons were cultured on a microfluidic device. At DIV4, chloramphenicol (CA) was added to either cell body or axonal chamber. After 30 min, BDNF was added to the axonal chamber. Neurons were stained and imaged at DIV5. **b** Representative images of axons. Axons were marked by Tau-1 immunostaining (scale bar, 200 μm). **c** The axonal length was measured from the exit border of microgrooves (dotted lines), including the main axons and branches. Data represent mean ± SEM (*N* = 4–5 replicates and *n* = 422–745 axons). n.s., not significant; ****P < 0.0001, as determined by Aligned ranks transformation ANOVA with Wilcoxon rank-sum test. **d** Representative images of primary hippocampal neurons expressing shRNA and mtIF3 (SR). Hippocampal neurons were co-transfected with shRNAs and overexpression vectors and then treated with BDNF at DIV1. The axonal length was measured at DIV3 (scale bar, 100 μm). TagRFP657 and mito-mTFP1 signals confirm co-transfection of shRNAs and overexpression vectors. White arrow head indicates the start point of axon and blue arrow head indicates the end point of axon. **e** Axon length was calculated by measuring TagRFP657 signals. Data represent mean ± SEM (*N* = 5 replicates and *n* = 50 neurons). Aligned ranks transformation ANOVA detected significant interactions of mtIF3 (SR) overexpression and BDNF on the axon length in mtIF3 shRNA groups (but not in control shRNA groups) (*P* = 0.0002). n.s., not significant; *P < 0.05, ****P < 0.0001, as determined by Wilcoxon rank-sum test

To determine whether local mitochondrial translation indeed impacts axonal growth, we applied CA to either cell bodies or axons of primary hippocampal neurons cultured in a microfluidic device (Fig. 5a). BDNF was subsequently added to the axonal chamber, and BDNF-induced axon growth was quantified accordingly. We found that selective inhibition of mitochondrial translation in axons, but not in cell bodies, suppressed BDNF-induced axon extensions (Fig. 5b, c). These data demonstrate that rapid axon extension by this trophic factor requires local mitochondrial translation in axons. Given that locally synthesized mtIF3 regulates the mitochondrial translation in axons, we reasoned that mtIF3 depletion would impair axonal extension. Indeed, transient expression of mtIF3 shRNA remarkably shortened axonal length compared to control shRNA and further silenced BDNF effects on axon development (Fig. 5d, e). mtIF3 overexpression partially restored axon length in mtIF3-depleted neurons in the absence of BDNF treatment and mtIF3 UTRs were dispensable for the overexpression effects. However, we found that BDNF-induced axon growth required mtIF3 expression through the mtIF3 UTRs (Fig. 5d, e). These results suggest that mtIF3 functions in both cell body and axon for axon development yet locally synthesized mtIF3 plays a more critical role in BDNF-induced axonal growth, likely via mtIF3-dependent mitochondrial translation and ATP generation in distal axons.

## Discussion

Local protein synthesis is a distinctive feature in highly polarized neurons and indispensable for the structural and functional maintenance of axons and dendrites such as neurite development, the guidance of growth cone, synaptic transmission, synaptic plasticity, branch formation, and regeneration [36–39]. The gene ontology analyses revealed high enrichment of synaptic proteins, cytoskeletal proteins, and ribosomal proteins in axonal translatome [11, 40]. Interestingly, it has also been identified that transcripts of nuclear-encoded mitochondrial proteins are abundant in developing and mature axons [10–14], implicating their local translation in sustaining mitochondrial function and axonal viability. Nonetheless, only a few studies have documented that local translation of nuclear-encoded mitochondrial proteins can affect mitochondrial function and axonal survival [41–44].

Here, we demonstrate that nuclear-encoded mtIF3 is locally translated in developing axons, thereby promoting axonal mitochondrial translation as assessed by our newly developed mito-riboBiFC sensor. Many studies have demonstrated that stationary mitochondria in axons fuel spatially restricted boundaries [1, 2], but what remains unsolved is how these stationary mitochondria are supported and maintained in the long term. Our results suggest that mitochondrial proteins may be replenished by enhanced mitochondrial translation via local protein synthesis in axons. We observed that mtIF3 depletion cancels out the upregulation of mitochondrial translation and ATP production upon BDNF stimulation. Lack of this local translation and adaptive control of mitochondrial function limits axonal development, validating its critical role in neuronal physiology. However, we also observed that the overexpression of mtIF3 per se did not affect mitochondrial functions. It has been recently shown that mitochondrial translation is synchronized and unidirectionally controlled by cytosolic translation [45]. Our observation consistently implicates that the enhancement of mitochondrial functions for local energy demands is accomplished by communicating with cytosolic translation. Therefore, our findings suggest that local translation in axons can be a crucial mechanism by which mitochondrial translation is regulated in mammalian neurons.

In the past decade, much effort has been made to develop tools for measuring or observing mitochondrial translation. For instance, biochemical detection of newly synthesized mitochondrial proteins has been widely used for studying mitochondrial translation [46–50]. However, a lack of appropriate imaging tools for mitochondrial translation has hindered assessing this subcellular event at single-cell levels. A recent study visualized mitochondrial translation using a non-canonical amino acid labeling in situ [51]. This method allows the detection of mitochondrial translation at a single-cell resolution, but its application is limited to fixed cells. Our study developed a new method designated as mito-riboBiFC to monitor mitochondrial translation in live cells. Mito-riboBiFC enables us to investigate mitochondrial translation on distinct spatiotemporal scales. Accordingly, it will be of great interest to determine how mitochondrial translation is regulated depending on their subcellular location or mitochondrial dynamics, especially in neurons where subcellular environment and energetic needs are spatially distinct.

Nonetheless, mito-riboBiFC has some limitations that should be improved in the future. These include relatively slow maturation kinetics of the mito-riboBiFC. Mitochondria are highly dynamic and heterogeneous in terms of their transport, membrane potential, and biogenesis. These mitochondrial events can occur on a relatively short timescale (e.g., a few seconds or minutes), compared to the folding and maturation time of the BiFC complex [52]. The employment of a new chromophore in BiFC imaging should improve the current temporal resolution of our mito-riboBiFC, better visualizing the rapid change in mitochondrial translation according to diverse mitochondrial dynamics.

## Conclusions

Our results provide new insights into understanding the adaptive regulation of mitochondrial physiology via local protein synthesis of a nuclear-encoded mitochondrial translation factor during axonal development. New imaging tools for the mitochondrial function should further dissect the molecular mechanisms underlying the spatiotemporal control of mitochondrial physiology and hint at novel therapeutic strategies to treat relevant neurodevelopmental diseases.

## Methods

### Animals

Embryos from pregnant mice (C57BL/6 J, Hyochang Science, Korea) were used for primary hippocampal neuron culture. All experimental procedures were conducted in accordance with protocols approved by Institutional Animal Care and Use Committee of Ulsan National Institute of Science and Technology (UNIST).

### Cell culture

#### Primary hippocampal neurons

Primary hippocampal neuron culture was processed as follows. In brief, hippocampi were dissected from E18 mouse embryos and they were washed with HBSS (Invitrogen). Hippocampi were digested by 0.025% trypsin (Invitrogen) and washed with trituration media (90% of Dulbecco Modified Eagle Medium and 10% fetal bovine serum, Invitrogen). Dissociated cells were seeded onto 50 μg/ml of poly-D-lysine (Sigma) coated culture dishes or coverslips. After the settlement of cells, neurons were maintained with neuronal culture media, which consists of Neurobasal media, GlutaMax, B27, and penicillin-streptomycin (Invitrogen). Neurons were transfected with lipofectamine 2000 (Invitrogen).

#### Cell lines

Neuro2A cell line was used for mito-riboBiFC experiments and purchased from ATCC. Neuro2A cells were maintained in culture media, which consists of Dulbecco Modified Eagle Medium and 10% fetal bovine serum, and 1% penicillin-streptomycin (Invitrogen). Using mycoplasma detection kit (Takara, 6601), we confirmed no contamination in Neuro2A cell line. PEI (Polysciences, 23966-1) or lipofectamine 2000 (Invitrogen) were used for transfecting constructs into Neuro2A cells. NIH/3 T3 cell line was purchased from ATCC and used for the modulation of mtIF3 expression. Cells were maintained in culture media, which consists of Dulbecco Modified Eagle Medium and 10% calf serum, and 1% penicillin-streptomycin (Invitrogen). Metafectene (Biontex) was used for the transfection of mtIF3 constructs.

### Separation of cell bodies and axons

To isolate the lysate of cell bodies and axons separately, neurons were seeded on the 6-well inserts with 3 μm pore size (SPL Life Sciences). Samples of cell bodies and axons were collected by scraping the upper and bottom sides of inserts. To treat cell bodies or axons separately with drugs, neurons were placed on microfluidic devices (Xona Microfluidics). Microfluidic devices were attached to a glass bottom dish (In Vitro Scientific, D60-30-1.5) for live cell imaging or 22 mm square coverslips (Globe Scientific, 1404-15) for fixed samples. We were aware that axons bundled with other axons might be influenced by cell-to-cell interactions. Unfortunately, we could not exclude this potential effect from other axons because our experiments using microfluidic devices required high density of the cells, and it was technically hard to find axons that did not touch other axons in the microfluidic devices. Despite this fact, however, all the control groups throughout the experiments (using microfluidic devices) were exposed to the same environment where axons were touching with other axons. Thus, we think that this potential effect by axon-to-axon interactions would not weaken our conclusion. Thirty nanograms per milliliter of BDNF (Sigma), 50 μg/ml of chloramphenicol (Sigma), 20 μM of anisomycin (Sigma), and 100 μg/ml of cycloheximide (Sigma) were used for drugs treatment.

### Vector preparation

For local protein synthesis assay, *pDendra2-C* vector (Evrogen) was modified: *5′UTR of mtIF3-2xPal-Dendra2-3′UTR of mtIF3*, *5′UTR of mtIF3-CDS of mtIF3-2xPal-Dendra2-3′UTR of mtIF3*, *CDS of mtIF3-3xPal-Dendra2*, and *5′UTR of GAPDH-2xPal-Dendra2-3′UTR of GAPDH*. To block the effect of diffusion, two repeats of the palmitoylation sequence were added. For mito-riboBiFC assay, *pcDNA6/V5-HisA* (Invitrogen) plasmid was modified: Neuro2A cDNA sequence of Mouse *Mrps6* (NM_080456.1), *Mrpl2* (NM_025302.4), *Mrps16* (NM_025440.3), and *Mrpl50* (NM_178603.4) were used to generate *MRPS6-VN172*, *MRPL2-VC155*, *MRPS16-VN172*, and *MRPL50-VC155* constructs. VC was fused to MRPL2 and MRPL50 by linker peptides: GSKQKVMNH. MRPS6 and MRPS16 were fused to VN by linker peptides: GSRSIAT. For the reduction of mtIF3 expression, *AAV-shRNA-ctrl* (Addgene, #85741) was modified: *pAAV2-Control-shRNA-TagRFP657*, *pAAV2-mtIF3-shRNA-TagRFP657*. For shRNA-resistant mtIF3, shRNA target site mutagenesis on mtIF3 was performed by overlap extension PCR. *pcDNA6/V5-HisA*, and *psi-Check2* plasmids were modified: *pcDNA6-5′UTR of mtIF3-CDS (SR) of mtIF3-3xFLAG-3′UTR of mtIF3*, *psi-Check2-SV40 promoter-3xFlag-CMV promoter-mito-mTFP1*, *psi-Check2-SV40 promoter-5′UTR of mtIF3-CDS (SR) of mtIF3-3xFLAG-3′UTR of mtIF3-CMV promoter-mito-mTFP1*, *psi-Check2-SV40 promoter-CDS (SR) of mtIF3-3xFLAG-CMV promoter-mito-mTFP1*, *psi-Check2-SV40 promoter-3xFlag-HSV-TK promoter-mCherry*, *psi-Check2-SV40 promoter-5′UTR of mtIF3-CDS (SR) of mtIF3-3xFLAG-3′UTR of mtIF3-HSV-TK promoter-mCherry*, and *psi-Check2-SV40 promoter-CDS (SR) of mtIF3-3xFLAG-HSV-TK promoter-mCherry.*

### Confocal microscopy and image analysis

All the images were taken using a confocal microscope (Zeiss LSM 780). Live cell imaging was performed in a live cell chamber that was maintained at 37 °C and 5% CO_2_ by a heating instrument. Definite Focus *z*-correction hardware was used to maintain the *z*-axis during the time lapse image. Orthogonal projection and image crop were processed in ZEN 3.1 (blue edition). Fluorescence signal intensity was quantified by ImageJ (NIH).

### Local protein synthesis assay

For local mRNA translation assay, Dendra2 fluorescence protein was conjugated with UTRs of mtIF3 or GAPDH: 5′UTR of mtIF3-Palmitoylation sequence-Dendra2-3′UTR of mtIF3, 5′UTR of mtIF3-CDS of mtIF3-Palmitoylation sequence-Dendra2-3′UTR of mtIF3, CDS of mtIF3-Palmitoylation sequence-Dendra2, and 5′UTR of GAPDH-Palmitoylation sequence-Dendra2-3′UTR of GAPDH. Primary hippocampal neurons were cultured on microfluidic devices and transfected with these vectors at DIV3 by using Lipofectamine 2000 (Invitrogen). Twenty-four hours after transfection, a protein synthesis assay was performed. The existing fluorescence of dendra2 (green) in the axonal tip was photoconverted into red fluorescence with 405 nm laser for 10 s, and newly synthesized green signals were measured for 90 min with 5 min time lapse image. Protein synthesis inhibitor, anisomycin (20 μM, Sigma) was used to confirm that the increased green signal was from de novo protein synthesis. BDNF (30 ng/ml, Sigma) and anisomycin were treated in an axonal chamber of microfluidic devices to locally induce or block protein synthesis. Images were acquired by using 488, 561 nm lasers.

### Live cell imaging

Before mito-riboBiFC imaging in Neuro2A cells, the culture medium was replaced and cells were incubated for 30 min. Twenty micrometers of puromycin (Sigma) was applied for 10 min. After puromycin treatment, 50 μg/ml of chloramphenicol (Sigma) was sequentially treated. To label mitochondria, mito-mTFP1 was also transfected. Images were acquired using 458, 514 nm lasers. To exclude and prevent FRET effect, we separately turned on and off two lasers (458 nm, 514 nm) in two different tracks. That is, only 514 nm laser was turned on when we detected mito-riboBiFC and mTFP1 was barely excited in this imaging setup. Therefore, our imaging procedure could suppress both bleed-through and FRET effect from mTFP1 signals. For ATP imaging, primary hippocampal neurons were cultured into microfluidic devices, which were attached on glass-bottom dishes. Neurons were transfected with shRNA vectors and overexpressing vectors at DIV1. At DIV3, images were acquired using 458, 514, and 633 nm lasers. CHX, BDNF, and CA were treated simultaneously and images were taken before drug treatment and after 90 min. Fluorescent intensity was measured from five mitochondria at the end of axons. The ratio of before and after drug treatment was averaged to measure the degree of mitochondrial translation. For mitochondrial ATP imaging, primary hippocampal neurons were transfected with genetically encoded FRET-based ATP indicator for mitochondria, mito-ATeam1.03, at DIV1 with shRNAs and overexpressing vectors by using Lipofectamine 2000 (Invitrogen). Images were taken at an emission of 475 nm and 527 nm with a 405 nm excitation laser. BDNF or cycloheximide was applied to the axonal chamber for 90 min. The increased ratio of FRET to CFP before and after drug treatment was calculated.

### Western blotting

Cells were lysed by using RIPA buffer (150 mM sodium chloride, 1% Triton X-100, 0.5% sodium deoxycholate and 0.1% sodium dodecyl sulfate). Proteins were separated by SDS-PAGE and transferred to PVDF membrane (Millipore) or NC membrane (GE healthcare). Membranes were blocked with 5% skim milk in TBST (10 mM Tris, 150 mM NaCl, 0.5% Tween 20) for 30–60 min. For immunoblotting, antibodies against mtIF3 (Sigma, HPA039791, polyclonal or ORIGENE, TA800421, monoclonal) and β-tubulin (Abcam, ab6046, polyclonal or Proteintech, 66240-1-lg, monoclonal) were incubated at 4 °C for overnight. Membranes were washed three times for 10 min with TBST and horseradish peroxidase-conjugated anti-rabbit or anti-mouse IgG secondary antibody (Jackson immunoresearch) was incubated for 1 h. Membranes were washed three times for 10 min with TBST and developed with ECL solution (Bio-Rad). For shRNA-resistant mtIF3, cells were washed twice and harvested with ice-cold PBS. Cells were collected by centrifugation at 2,500 g for 5 min at 4 °C. Then, cells were lysed by RIPA buffer (50 mM Tris-Cl pH 8.0, 150 mM NaCl, 5 mM EDTA, 10% NP-40, 0.5% Sodium deoxycholate, and 1 mM PMSF). The whole cell lysate was used for western blot. Images of full western blots can be found in Additional file 5.

### RT-PCR

Total RNA of the cell body and axon fractionations was isolated with PicoPure RNA isolation kit (Applied Biosystems). Two hundred nanograms of RNA was subjected to RT-PCR by using High Capacity RNA-to-cDNA kit (Life Technologies). Raw gel images were shown in Additional file 6. The primers used for PCR:

forward 5′-GAGAGCAGATCCACCAGGAG-3′ and

reverse 5′-CTGTTTCCGTCGTCGTCTTT-3′ for mtIF3;

forward 5′-ACCAACTGGGACGACATGGAGAAGA-3′ and

reverse 5′-CGTTGCCAATAGTGATGACCTGGCC-3′ for β-actin;

forward 5′-GGACGACATGGAGAAGATCTGGCAC-3′ and

reverse 5′-CCGGACACCGGAACCGCTCATTG-3′ for γ-actin.

### Immunostaining

For primary hippocampal neurons, cells were rinsed with PBS and fixed with 4% PFA for 10 min. Cells were permeabilized with PBST (PBS with 0.2% Triton X-100) for 10 min and blocked with 1% BSA in PBST for 30 min. Primary antibodies were incubated at 4 °C overnight. Antibody against Tau1 (Millipore, MAB3420, monoclonal) was used for immunostaining. The cells were washed three times with PBS and incubated with Alexa Fluor secondary antibodies (Invitrogen). Then these cells were washed three times with PBS and coverslips were mounted on slide glasses. Images were taken by using LSM780 confocal microscopy. For Neuro2A, cells were washed with ice-cold PBS followed by fixation using 4% PFA/sucrose for 20 min. After 3 times washing with PBS, cells were permeabilized with PBST (PBS with 0.5% Triton X-100) for 15 min and blocked with 1% BSA in PBS for 1 h. Primary antibodies were incubated at 4 °C overnight. Cells were washed 3 times with PBS and incubated with Alexa Fluor secondary antibodies (Invitrogen) for 1 h at room temperature. Cells were washed three times with PBS and coverslips were mounted on slide glasses. Antibodies against GFP (Abcam, ab6556, polyclonal), MT-CO1 (Abcam, ab14705, monoclonal), and Flag (Sigma, F1804, monoclonal) were used for immunostaining. Images were acquired by using 405, 458, 488, 561, 594, and 633 nm lasers.

### Statistical analysis

Statistical analyses were performed by using Prism software (GraphPad Software) or R (version 3.6.1) with ARTool library [53, 54]. All the values were presented with mean ± SEM. Shapiro-Wilk test for normality (*P* < 0.05) or *F*-test and Brown-Forsythe test for equal variance (*P* < 0.05) were performed for each dataset. Two-way ANOVA with Holm-Sidak’s test (repeated measures), aligned ranks transformation ANOVA with Wilcoxon signed-rank test (repeated measures), or Wilcoxon rank-sum (non-repeated measures) was used to determine statistical differences between the groups. *P* < 0.05 was considered statistically significant. *P < 0.05, **P < 0.01, ***P < 0.001, ****P < 0.0001. All the statistical analysis can be found in Additional file 7.

## Supplementary Information

**Additional file 1: Fig. S1.** mtIF3-Dendra2 is localized to mitochondria. **a** Schematic illustration of mtIF3 coding sequence. mtIF3 has mitochondrial targeting sequence in N-terminal domain (131 a.a.). **b** Primary hippocampal neurons were transfected with Dendra2 vectors. Mitochondria were labeled with mitochondrial-targeted mCherry (mito-mCherry) transfection. CDS of mtIF3 led to mitochondrial localization of Dendra2 (scale bar, 10 μm). **c** Original image of the area used for the kymograph. These images were taken at 90-minute timepoint after drug treatment (scale bar, 10 μm). Axons were imaged from axonal chamber of microfluidic devices.

**Additional file 2: Fig. S2.** Schematic illustration of mito-riboBiFC analysis. Translating ribosomal complex exhibits active dynamics. During the elongation, ribosomal subunits consistently rotate, which results in low intensity mito-riboBiFC. To freeze translating mitochondrial ribosomes, we treated chloramphenicol that inhibits the formation of peptide bond. Non-rotated ribosomal complex is expected to show high BiFC signal. 90 minutes after the treatment of chloramphenicol, we compared the intensity of mito-riboBiFC before and after chloramphenicol treatment. Because highly translating mRNA binds to more ribosomes, we could detect higher signal increase in actively translating mRNA.

**Additional file 3: Fig. S3.** Modulation of mtIF3 expression level. **a, b** mtIF3 level was reduced by RNA interference with shRNA. By performing western blot, we verified the successful reduction of mtiF3 48 h after incubation of shRNA in NIH/3T3 cells. The level of mtIF3 was decreased about 50% compared with control group. The mtIF3 expression level was normalized to β-tubulin level. Data represent mean ± SEM (N = 5 independent experiments). *P < 0.05 as determined by unpaired Welch’s t-test. **c** Sequence of shRNA-resistant (SR) mtIF3. 7 nucleotides of mtIF3 CDS were mutated at mtIF3 shRNA targeting site. **d** Co-expression of mtIF3 (SR) with shRNA analyzed by western blot. shRNA and 5’UTR-mtIF3 (SR)-3xFLAG-3’UTR plasmids were co-transfected for 72 h in Neuro2A cells. **e** Relative expression level of mtIF3 with UTRs or without UTRs analyzed by western blot. Plasmid of mtIF3 (SR) with UTRs or without UTRs was transfected for 72 h in Neuro2A cells. The gradually increasing amount of mtIF3 (SR)-3xFLAG plasmid was used for transfection to compare with UTR-dependent expression level of mtIF3. * indicates 3xFlag-tagged mtIF3 (SR) and ** indicates endogenous mtIF3. β-tubulin was used for loading control. **f, g** Dual promoter constructs and representative images of mtIF3 with mito-mTFP1 or mCherry in Neuro2A cells (Scale bar, 5 μm).

**Additional file 4: Fig. S4.** Blocking mitochondrial translation decreases mitochondrial ATP level. Treatment of chloramphenicol caused the rapid decrease of FRET intensity. Primary hippocampal neurons were transfected with Mt-ATeam1.03 at DIV2 and images were taken every 5 minutes for 90 minutes at DIV3. After the first image was taken, chloramphenicol was treated during the whole experimental time without washing. The values are presented as mean ± SEM and statistical significance was tested between 5 minutes before treatment and 0 minute after treatment using paired t-test. N = 10 cells from 3 independent experiments. ***P < 0.001.

**Additional file 5.** Raw images of the immunoblots in Fig. S3.

**Additional file 6.** Raw images of RT-PCR results in Fig. 1a.

**Additional file 7.** Statistical analysis. Summary of all statistical analysis in this article.

## Data Availability

All data generated or analyzed during this study are included in this published article and its supplementary information files.

## Abbreviations

mtIF3: Mitochondrial initiation factor 3
BDNF: Brain-derived neurotrophic factor
Mitoribosomes: Mitochondrial ribosomes
BiFC: Bimolecular fluorescence complementation
UTRs: Untranslated regions
CDS: Coding sequence
MRPL2: Mitochondrial ribosomal protein L2
MRPS6: Mitochondrial ribosomal protein S6
CA: Chloramphenicol
shRNA: Short hairpin RNA
SR: shRNA-resistant
CHX: Cycloheximide
VN: mVenus into N-terminal
VC: mVenus into C-terminal
FRET: Fluorescence Resonance Energy Transfer
