# Supplementary figures and images for: mtIF3 is locally translated in axons and regulates mitochondrial translation for axonal growth
Source: BMC Biol. 2022 Jan 7;20:12. doi: 10.1186/s12915-021-01215-w (PMC8742369; doi:10.1186/s12915-021-01215-w)

**a**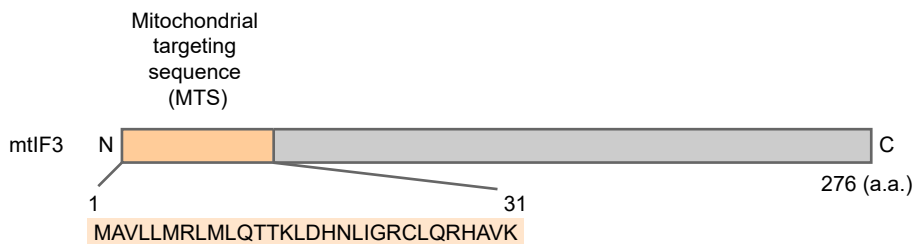**b**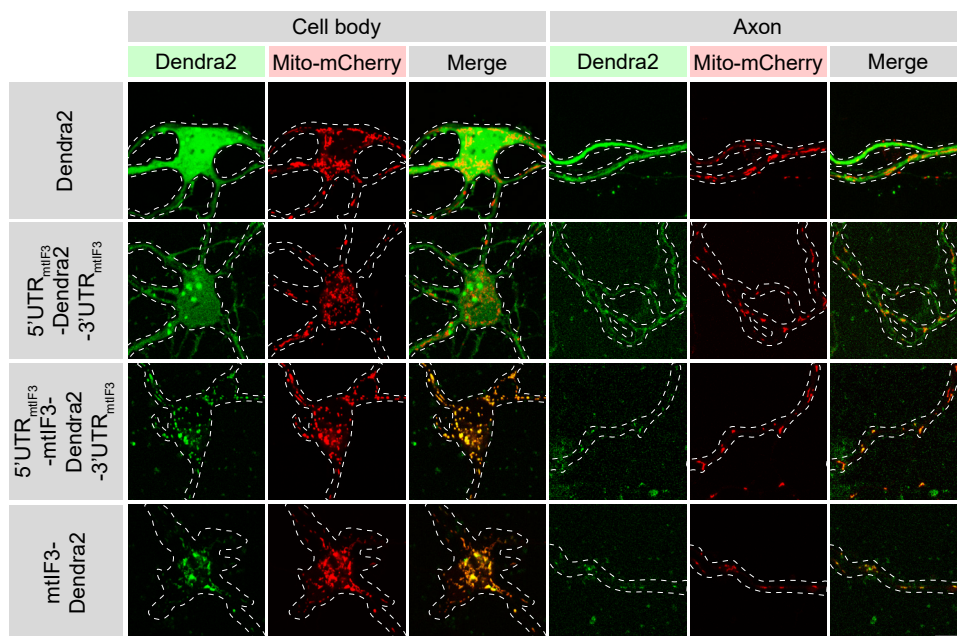**c**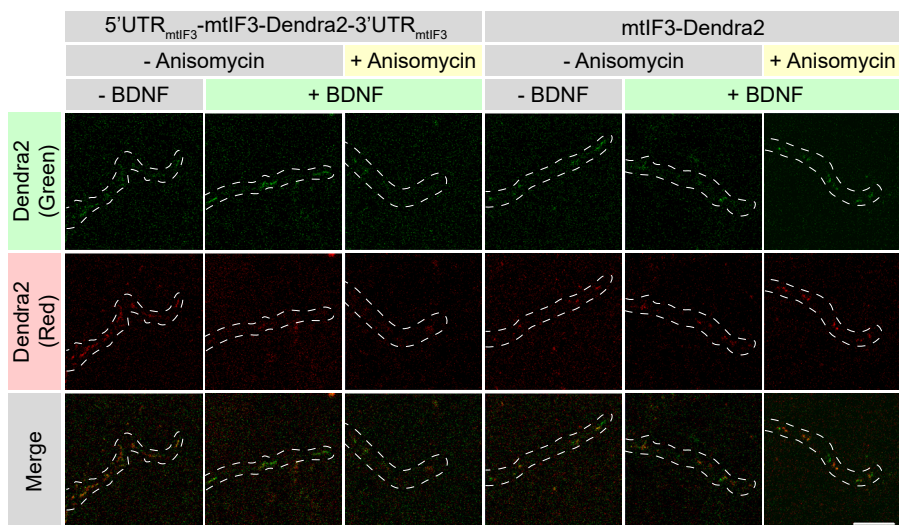

Supplement: Supplementary file 1 — Additional file 1: Fig. S1. mtIF3-Dendra2 is localized to mitochondria. a Schematic illustration of mtIF3 coding sequence. mtIF3 has mitochondrial targeting sequence in N-terminal domain (131 a.a.). b Primary hippocampal neurons were transfected with Dendra2 vectors. Mitochondria were labeled with mitochondrial-targeted mCherry (mito-mCherry) transfection. CDS of mtIF3 led to mitochondrial localization of Dendra2 (scale bar, 10 μm). c Original image of the area used for the kymograph. These images were taken at 90-minute timepoint after drug treatment (scale bar, 10 μm). Axons were imaged from axonal chamber of microfluidic devices. [file 12915_2021_1215_MOESM1_ESM.pdf]

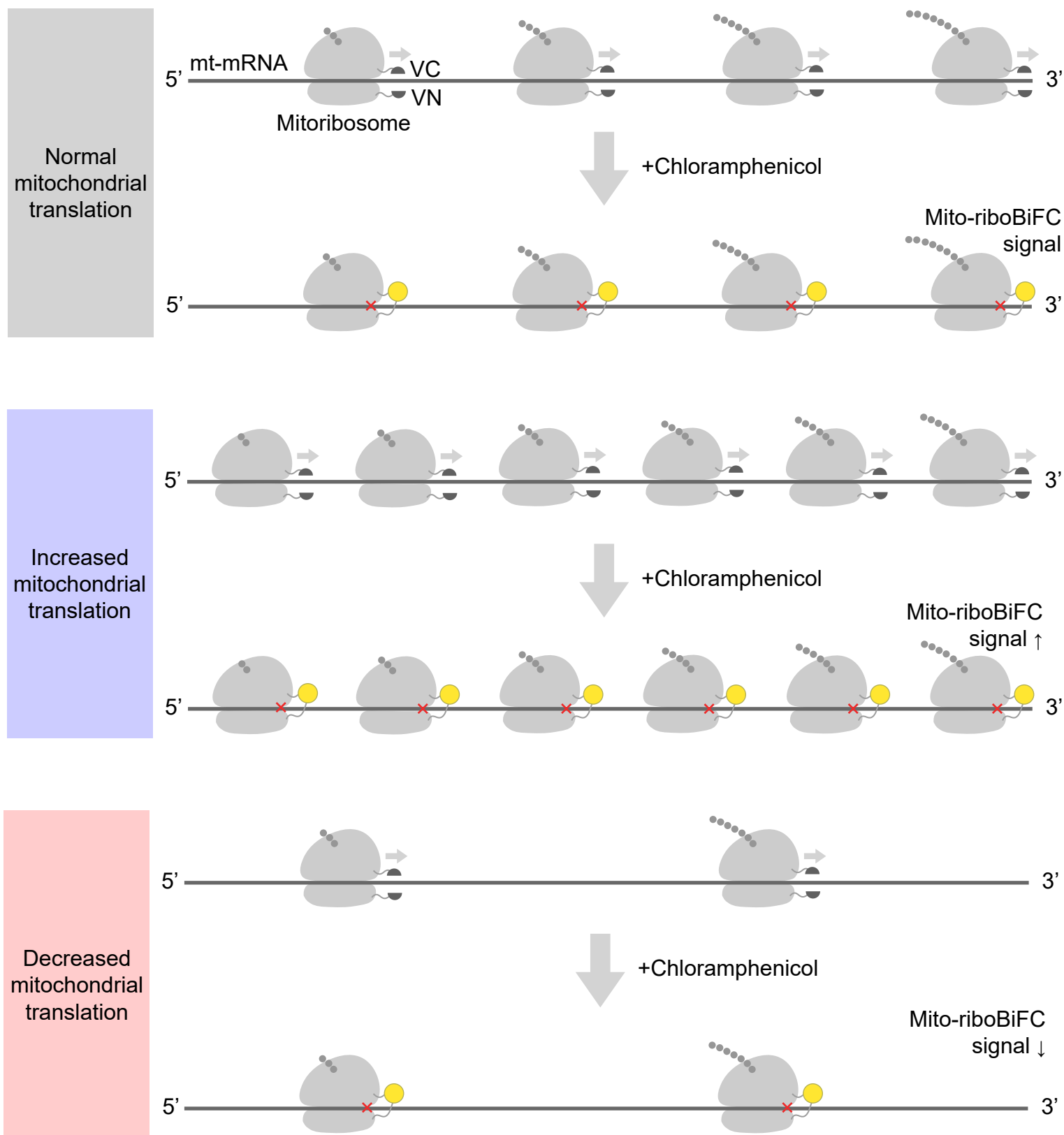

Supplement: Supplementary file 2 — Additional file 2: Fig. S2. Schematic illustration of mito-riboBiFC analysis. Translating ribosomal complex exhibits active dynamics. During the elongation, ribosomal subunits consistently rotate, which results in low intensity mito-riboBiFC. To freeze translating mitochondrial ribosomes, we treated chloramphenicol that inhibits the formation of peptide bond. Non-rotated ribosomal complex is expected to show high BiFC signal. 90 minutes after the treatment of chloramphenicol, we compared the intensity of mito-riboBiFC before and after chloramphenicol treatment. Because highly translating mRNA binds to more ribosomes, we could detect higher signal increase in actively translating mRNA. [file 12915_2021_1215_MOESM2_ESM.pdf]

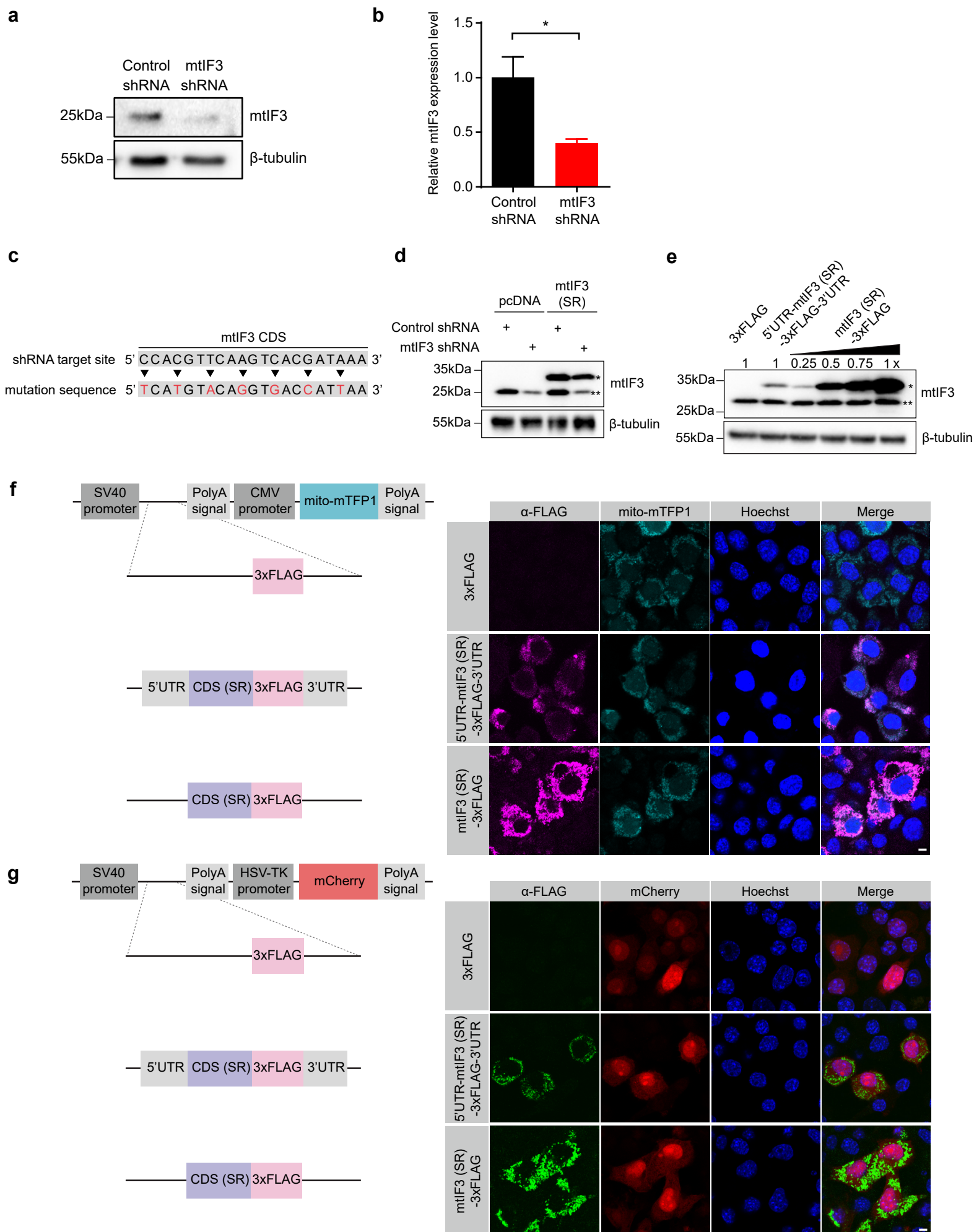

Supplement: Supplementary file 3 — Additional file 3: Fig. S3. Modulation of mtIF3 expression level. a, b mtIF3 level was reduced by RNA interference with shRNA. By performing western blot, we verified the successful reduction of mtiF3 48 h after incubation of shRNA in NIH/3T3 cells. The level of mtIF3 was decreased about 50% compared with control group. The mtIF3 expression level was normalized to β-tubulin level. Data represent mean ± SEM (N = 5 independent experiments). *P < 0.05 as determined by unpaired Welch’s t-test. c Sequence of shRNA-resistant (SR) mtIF3. 7 nucleotides of mtIF3 CDS were mutated at mtIF3 shRNA targeting site. d Co-expression of mtIF3 (SR) with shRNA analyzed by western blot. shRNA and 5’UTR-mtIF3 (SR)-3xFLAG-3’UTR plasmids were co-transfected for 72 h in Neuro2A cells. e Relative expression level of mtIF3 with UTRs or without UTRs analyzed by western blot. Plasmid of mtIF3 (SR) with UTRs or without UTRs was transfected for 72 h in Neuro2A cells. The gradually increasing amount of mtIF3 (SR)-3xFLAG plasmid was used for transfection to compare with UTR-dependent expression level of mtIF3. * indicates 3xFlag-tagged mtIF3 (SR) and ** indicates endogenous mtIF3. β-tubulin was used for loading control. f, g Dual promoter constructs and representative images of mtIF3 with mito-mTFP1 or mCherry in Neuro2A cells (Scale bar, 5 μm). [file 12915_2021_1215_MOESM3_ESM.pdf]

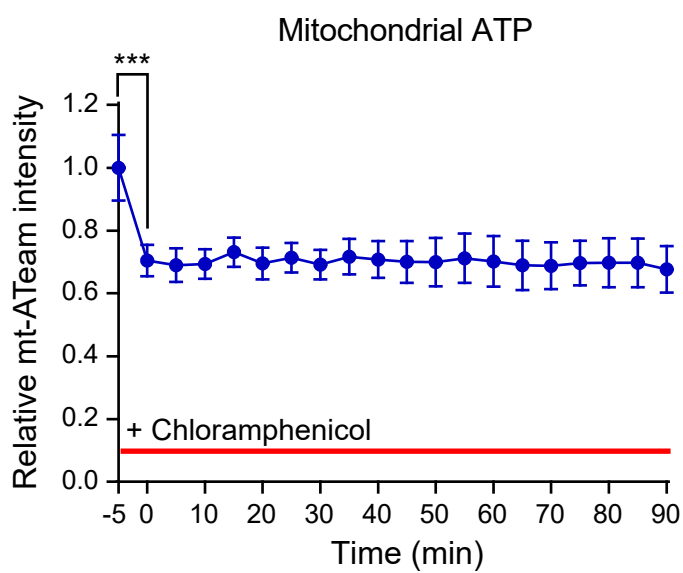

Supplement: Supplementary file 4 — Additional file 4: Fig. S4. Blocking mitochondrial translation decreases mitochondrial ATP level. Treatment of chloramphenicol caused the rapid decrease of FRET intensity. Primary hippocampal neurons were transfected with Mt-ATeam1.03 at DIV2 and images were taken every 5 minutes for 90 minutes at DIV3. After the first image was taken, chloramphenicol was treated during the whole experimental time without washing. The values are presented as mean ± SEM and statistical significance was tested between 5 minutes before treatment and 0 minute after treatment using paired t-test. N = 10 cells from 3 independent experiments. ***P < 0.001. [file 12915_2021_1215_MOESM4_ESM.pdf]

Figure S3a Raw Images

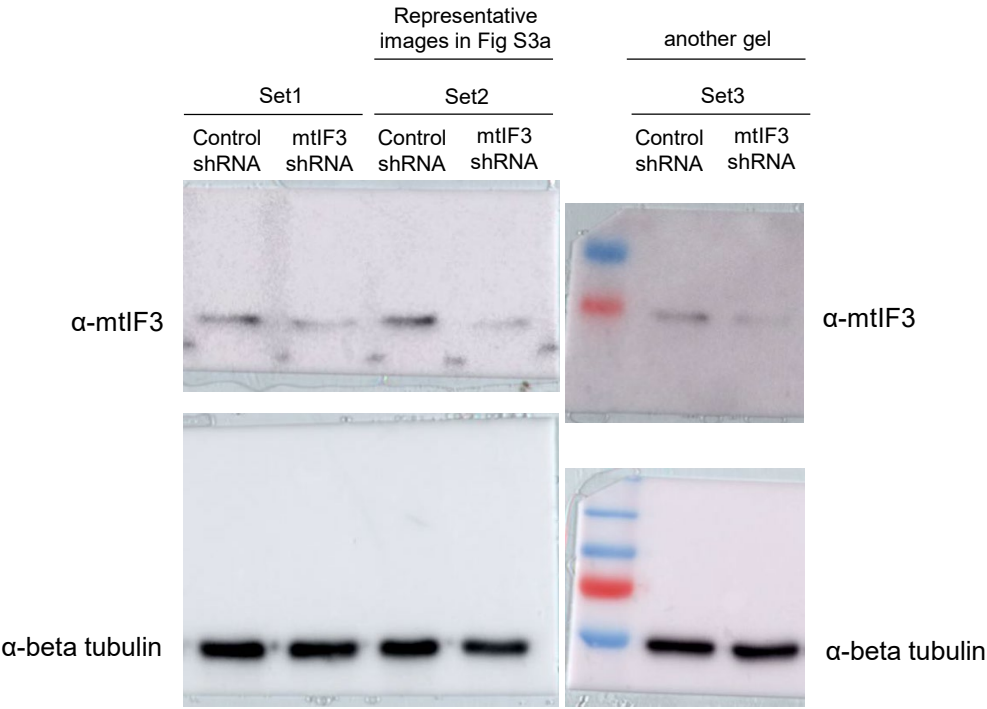

Figure S3d Raw Images

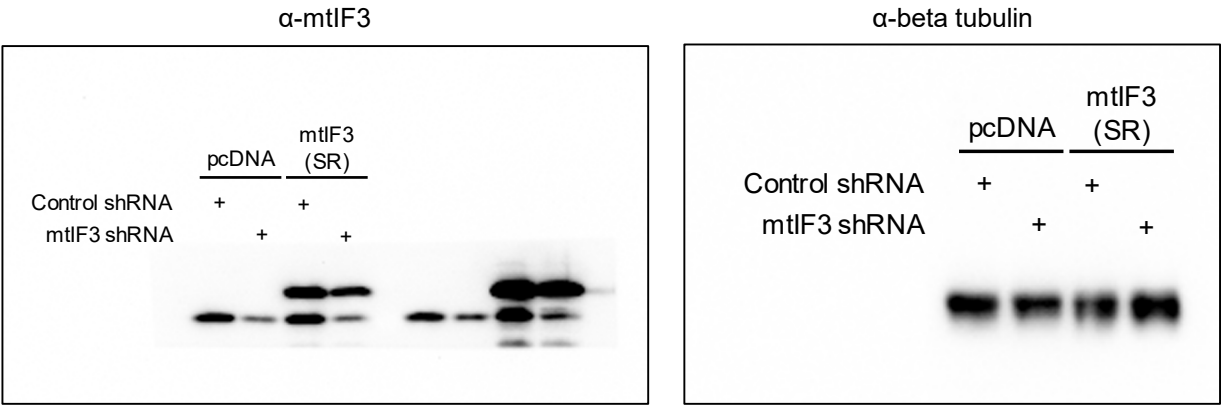

Figure S3e Raw Images

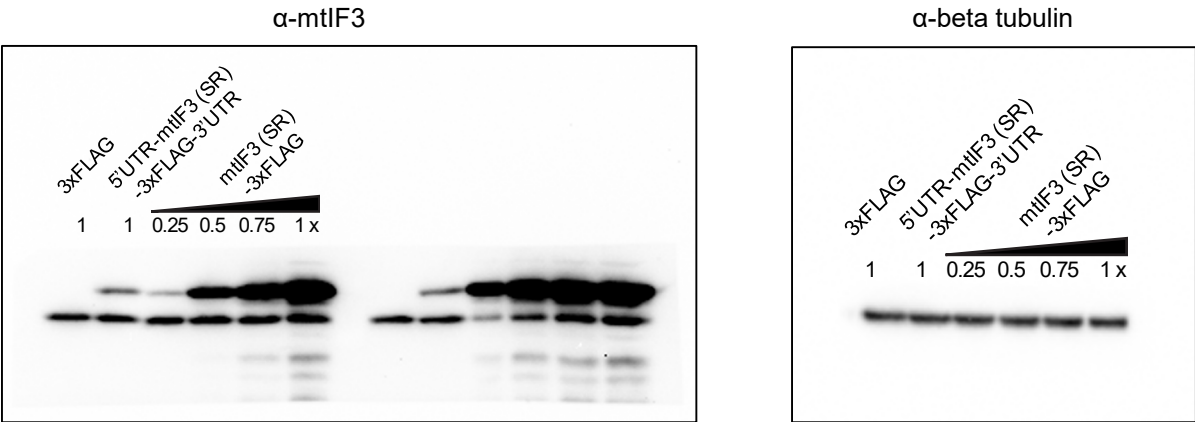

Supplement: Supplementary file 5 — Additional file 5. Raw images of the immunoblots in Fig. S3. [file 12915_2021_1215_MOESM5_ESM.pdf]

Figure 1a Raw Gel Image

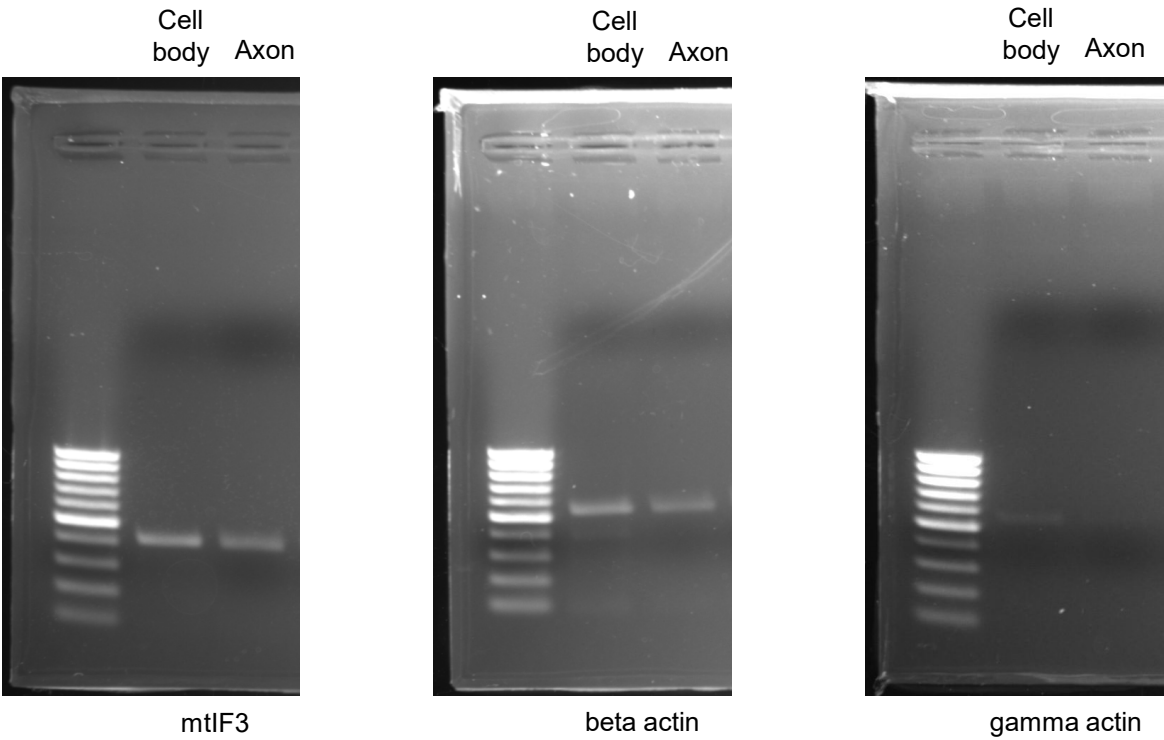

Supplement: Supplementary file 6 — Additional file 6. Raw images of RT-PCR results in Fig. 1a. [file 12915_2021_1215_MOESM6_ESM.pdf]
